# A multi-year study monitoring the cadmium content in the tissues of lambs and sheep sampled in the Czech Republic between 2001 and 2022

**DOI:** 10.17221/45/2024-VETMED

**Published:** 2024-09-25

**Authors:** Zdenka Svobodova, Jiri Drapal, Veronika Vlasakova, Danka Harustiakova, Josef Illek, Martin Svoboda

**Affiliations:** ^1^Department of Animal Protection and Welfare and Veterinary Public Health, Faculty of Veterinary Hygiene and Ecology, University of Veterinary Sciences Brno, Brno, Czech Republic; ^2^Central Veterinary Administration of the State Veterinary Administration, Prague, Czech Republic; ^3^Faculty of Science, RECETOX, Masaryk University, Brno, Czech Republic; ^4^Large Animal Clinical Laboratory, Faculty of Veterinary Medicine, University of Veterinary Sciences Brno, Brno, Czech Republic; ^5^Ruminant and Swine Clinic, Faculty of Veterinary Medicine, University of Veterinary Sciences Brno, Brno, Czech Republic

**Keywords:** heavy metal, kidney, lamb, liver, sheep

## Abstract

Cadmium is one of the most important environmental contaminants. Animals grazing on natural pastures are particularly exposed to cadmium. Sheep are mostly reared in extensive and grazing systems. Therefore, sheep may accumulate larger amounts of cadmium compared to other ruminant species and are a good indicator of exposure to cadmium contamination in a given area. The determination of cadmium concentrations in the muscles, livers and kidneys of lambs and sheep was carried out in the Czech Republic during the period 2001–2022. The average cadmium content in the livers and kidneys of all the lambs was 0.037 ± 0.006 and 0.061 ± 0.013 mg.kg^–1^, respectively. The average cadmium content in the livers and kidneys of all the sheep was 0.319 ± 0.047 and 1.255 ± 0.204 mg.kg^–1^, respectively. The maximum limit for human consumption was not exceeded in any of the 24 samples in the lambs but was exceeded in 5 of 33 liver samples and 12 of 33 kidney samples in the adult sheep. The average value of cadmium in the muscles of the lambs and sheep was 0.004 ± 0.001 mg.kg^–1^. The maximum limit for human consumption was not exceeded in any muscle sample. The cadmium content in both the liver and in the kidney differs significantly between the lambs and sheep, being higher in the sheep (*P* < 0.001 for both comparisons). No statistically significant trend of a decrease in the cadmium content in the lamb and sheep tissues was found during the observed time (*P* > 0.05). We can sum up that there is an evident need for further monitoring of the cadmium concentration in lamb and sheep tissues in the Czech Republic.

Cadmium (Cd) is one of the most important environmental contaminants. During the 20^th^ century, there was a significant increase in the content of cadmium in agricultural soil and its products (Linden et al. 2003).

The source of cadmium in the environment is mainly its anthropogenic use, which is associated with many different activities, such as metal mining and the industrial processing of metals (MacLachlan et al. 2013). A significant source of air contamination with cadmium is the burning of fossil fuels (Steinnes 1984). The contamination of the soil is caused by cadmium falling from the air, but also by the use of phosphate fertilisers, which can be contaminated with cadmium (Morcombe et al. 1994a; Jarup 2003). Sewage sludge is also considered an important sources of Cd contamination (Heffron et al. 1980; Patrick 2003).

Long-term exposure to low concentrations of environmental cadmium can have a negative effect on the health of both livestock and humans consuming products derived from these animals (Grawe et al. 1997).

Of particular importance is the fact that cadmium can act as a multi-tissue carcinogen (Waalkes 2000) and is a known human carcinogen (Filipic et al. 2006). In people chronically exposed to cadmium, the risk of bone fractures, cancer, kidney dysfunction and hypertension increases (Mendez-Armenta and Rios 2011).

Cadmium accumulates in the soil, plants and water, even in areas that are far from the primary source of emissions (Steinnes 1989; Dobrzanski et al. 1996; Rogowska et al. 2008). Animals grazing on natural pastures are particularly exposed to cadmium. Sheep are mostly reared in extensive and grazing systems; therefore, sheep may accumulate larger amounts of cadmium compared to other ruminant species (Rogowska et al. 2008). Sheep can therefore be a good indicator of exposure to cadmium contamination in a given area (Bilandzic et al. 2010; Tunegova et al. 2016)

Sheep meat and sheep offal can form a significant part of the human diet and, thus, contribute to the overall exposure of consumers to cadmium. It is therefore necessary for the safety of consumers to carry out regular checks of the cadmium content in the tissues of these animals (Psenkova and Toman 2020).

As sheep farming conditions can change over time, it is important to periodically update our information on the sheep tissue content (MacLachlan et al. 2016).

The aim of this article was to process and present the results of monitoring the content of cadmium in the tissues of lambs and sheep carried out in the Czech Republic in the period from 2001 to 2022.

## MATERIAL AND METHODS

Veterinary inspectors of the Czech State Veteri-nary Administration selected randomly slaughtered lambs and sheep for tissue sampling at various slaughterhouses in the Czech Republic.

This monitoring was carried out within the national plan for monitoring of residues and contaminants in accordance with the Council Directive 96/23/EC (1996) in the years 2001–2021. Samples included the muscle (lean meat), any part of the liver (sample of at least 0.5 kg) and the whole kidney.

The lambs are slaughtered at an average age of approximately 6 months and sheep at 2–3 years of age.

Determination of the cadmium in the mentioned tissues was carried out in the laboratories of the State Veterinary Institutes in the Czech Republic. All the laboratories were accredited according to EN ISO/IEC 17025.

Electrothermal atomic absorption spectrometry with Zeeman correction (ET-AAS, or GF-AAS) or, in some cases, inductively coupled plasma mass spectrometry (ICP-MS) were used for these analyses.

The detailed procedure used in the processing and analysis of the samples was identical to the methodology presented in the publication of authors Svoboda et al. (2020).

### Statistical analysis

The number of liver and kidney samples analysed from 2001 to 2022 was 57 (24 lamb, 33 sheep), and the number of muscle samples was 51 (22 lamb, 29 sheep). The differences in the cadmium content between the liver, kidney and muscle and between the lambs and sheep were analysed using a repeated measures analysis of variance (ANOVA) followed by multiple *t*-tests with Bonferroni correction to identify the differences between the sample groups. The number of samples in the individual years ranged from 0 to 6, the annual mean values were used to evaluate the time trends of the cadmium concentration using Pearson’s correlation coefficient. *P* < 0.05 was considered significant in all the tests. Data manipulation and statistical analysis were performed using Statistica v14 (TIBCO Software Inc., Palo Alto, USA).

## RESULTS

The cadmium content found in the livers varied between 0.008 and 0.145 mg.kg^–1^ in the lambs and did not exceed the maximum limit (ML) for human consumption in any sample (ML for cadmium in liver: 0.5 mg.kg^–1^). The cadmium concentration measured in the sheep was higher, ranging from 0.079 to 1.430 mg.kg^–1^, and the ML was exceeded in 5 of 33 samples. The cadmium concentrations in the kidneys ranged from 0.011 and 0.350 mg.kg^–1^ in the lambs and from 0.257 to 6.023 mg.kg^–1^ in the sheep. The ML for human consumption (ML for cadmium in kidney: 1 mg.kg^–1^) in the lambs was not exceeded in any sample, but, in the sheep, it was exceeded in 12 of 33 samples. A cadmium concentration of 0.002–0.007 mg.kg^–1^ was found in the muscle. The ML for human consumption (ML for cadmium in muscle: 0.05 mg.kg^–1^) was not exceeded in any muscle sample, neither in the lambs nor in the sheep (Table 1).

**Table 1:** Cadmium content in the tissues of the lambs and sheep (mg.kg^–1^)

Animal	Tissue	*N*	Median	Mean ± SE	Min–max
Lamb	liver	24	0.033	0.037 ± 0.006^A,a^	0.008–0.145
	kidney	24	0.048	0.061 ± 0.013^A,a^	0.011–0.350
	muscle	22	0.005	0.004 ± 0.001^B,a^	0.002–0.005
Sheep	liver	33	0.241	0.319 ± 0.047^A,b^	0.079–1.430
	kidney	33	0.820	1.255 ± 0.204^B,b^	0.257–6.023
	muscle	29	0.005	0.004 ± 0.001^C,a^	0.002–0.007

^A–C^The cadmium concentration in the different tissues followed by the same uppercase superscript did not differ significantly (separately for lambs and sheep); ^a,b^The cadmium concentration in the lamb and sheep followed by the same lowercase superscript did not differ significantly (separately for liver, kidney and muscle)

The cadmium concentration was significantly affected by the age category (lambs/sheep) and by the tissue (repeated measures ANOVA: the effect of the tissue: F(2,98) = 26.254, *P* < 0.001; the effect of the age category: F(1,49) = 24.978, *P* < 0.001). The cadmium concentration was significantly higher in the livers and kidneys than in the muscles, both in the lambs and in sheep. In the sheep, significant differences in the cadmium concentration were found between all the tissues, being the highest in the kidneys and the lowest in the muscles (*P* < 0.001 in all three comparisons). In the lambs, significant differences in cadmium concentration were found between the livers and muscles (*P* < 0.001) and between the kidneys and muscles (*P* = 0.004). No difference in the cadmium content was found between the livers and kidneys (*P* = 0.078) (Table 1).

The cadmium content in both the livers and in the kidneys differs significantly between the lambs and sheep, being higher in the sheep (*P* < 0.001 for both comparisons; Table 1).

No statistically significant trend of a decrease in the cadmium content in the lamb and sheep tissues was found during the observed time period (the correlation coefficient between the mean cadmium concentration and year varied from –0.438 to 0.327, *P* > 0.05 in all the cases).

## DISCUSSION

The toxic effect of cadmium in ruminants is less often mentioned. The toxic effects of cadmium in ruminants have only been described experimentally, but there are no publications describing the naturally occurring intoxication of farmed ruminants.

Kidney damage, which is considered a typical feature of chronic cadmium intoxication, generally occurs at a cadmium concentration in the kidney tissue of 80–200 mg.kg^–1^ wet weight (Shore and Douben 1994; Lopez Alonso et al. 2000).

According to Puls (1994), the following is the categorisation of the cadmium content in kidney tissues with respect to animal health: low (0.05–1.5 mg.kg^–1^), high (5.0–36 mg.kg^–1^) or toxic (100–250 mg.kg^–1^).

The concentrations that we measured in the kidneys belong to the category of low values and thus mean a minimal risk from the point of view of the sheep’s health.

In the following section, we present examples of the results of monitoring the cadmium content in sheep tissues carried out in the countries of the European Union.

In a Spanish study, the authors of Pereira et al. (2021) stated that the mean cadmium concentrations were higher in the kidneys (751 ± 42 μg.kg^–1^) than in the livers (149 ± 10 μg.kg^–1^), and the mean concentrations in both organs were two orders of magnitude higher than in the muscles (5.2 ± 1.8 μg.kg^–1^). They also found that cadmium in 24% of the kidney samples exceeded the limits established by the EU.

In a Greek study, the authors Zantopoulos et al. (1999) found average cadmium concentrations in the livers of 0.224 mg.kg^–1^, in the kidneys of 0.830 mg.kg^–1^. The maximum value was found in the kidneys and was greater than 3 mg.kg^–1^.

Another example is the Croatian study by Bi-landzic et al. (2010), who found an average concentration of cadmium in the kidney tissues of 0.235 mg.kg^–1^. The maximum value in the kidney tissues was 3.56 mg.kg^–1^. The occurrence of kidney Cd concentrations exceeding the European maximum permitted concentrations (1 mg.kg^–1^) was found in 16% of the sheep.

In a Swedish study by the author Jorhem (1999), the average concentrations of cadmium in the sheep muscles were found to be 0.003 4 ± 0.004 3 mg.kg^–1^. The maximum concentration of cadmium in the muscles was 0.019 mg.kg^–1^. The average cadmium concentrations were found in the sheep kidneys at 1.0 ± 0.92 mg.kg^–1^, while the maximum value was 4.5 mg.kg^–1^.

In a study carried out in Poland (Rudy 2009), the average concentrations of cadmium in the sheep muscles were found to be 0.017 mg.kg^–1^, with a maximum value of 0.032 mg.kg^–1^. The average cadmium concentrations of 0.335 mg.kg^–1^, with the highest value of 0.498 mg.kg^–1^, were found in the livers of these sheep.

The Slovak authors Psenkova and Toman (2020) conducted their monitoring in the area with a slightly disturbed environment in the Western part of Slovakia, characterised by the load of energy emissions produced by the thermal power station in this area.

The average cadmium concentrations of 0.12 ± 0.05 mg.kg^–1^ were found in the livers. The maximum value in the livers reached 0.15 mg.kg^–1^. The average cadmium concentrations of 0.64 ± 0.15 mg.kg^–1^, with a maximum value of 0.84 mg.kg^–1^, were determined in the kidneys.

Australia is a country with significant sheep breeding. In a study by MacLachlan et al. (2016), the highest cadmium concentrations were found in the kidneys (mean 0.853 mg.kg^–1^) followed by the livers (mean 0.28 mg.kg^–1^). Ninety-one percent (91%) of the muscle samples were below the detection limit. The average concentration of cadmium in the muscles was 0.003 5 mg.kg^–1^.

In the available literature, we found many different results between the individual authors. In addition to the main factor, which is that individual areas differ from each other in the level of environmental cadmium contamination, there are several other reasons that can cause differences between the individual experimental studies.

For example, the cadmium content in the soil is only one of the factors that can affect the amount of cadmium in plants. Other factors include the organic matter content in the soil, the pH of the soil, whether there is crop rotation, and the content of other nutrients in the soil. The amount of cadmium that gets from the soil to the plants can also be influenced by the plant species or its variety (Eriksson et al. 1996; Hamon et al. 1998; Kirkham 2006; Phillips and Tudoreanu 2011).

For instance, Morcombe et al. (1994b) found that sheep grazing in pastures on acidic soils and soils with a sandy-textured surface had higher Cd concentrations in the kidneys than sheep grazing in pastures on more alkaline soils with a clay-textured surface.

Our results and the results of other authors show a relatively high deviation between the measured values. This indicates high inter-individual variability in the tissue cadmium content. The following explanation can be mentioned to explain this phenomenon:

The bioavailability and accumulation of cadmium in the animal organism can be affected by individual factors such as the nutritional status, multiple pregnancies and health status (European Food Safety Authority 2009).

For example, the intestinal absorption of cadmium is affected by the iron status, as cadmium and iron are absorbed from the intestine probably by a similar mechanism (Ohrvik et al. 2007). The authors Smith and White (1997) found that an increased supply of Mo and S in the diet reduced the accumulation of cadmium in the tissues of sheep.

For example, Houpert et al. (1997) found that there is a higher bioavailability of cadmium in lactating sheep compared to non-lactating sheep.

As part of our monitoring, higher concentrations of cadmium were found in the livers and kidneys compared to the muscle tissues. This is in line with other authors, for example, the authors Pope and Rall (1995) state that cadmium is primarily stored in the liver and kidneys, which make up half of the total cadmium reserves in the body.

In our study, we also found that the cadmium concentrations in the livers and the kidney are higher in the sheep compared to the lambs. This is in agreement with other studies, for example, the study by Australian authors MacLachlan et al. (2016) who also found age-related differences in the tissue concentrations of cadmium in sheep. An important factor contributing to this phenomenon is the fact that cadmium absorbed by sheep is poorly excreted with tissue elimination half-lives typically greater than 1 000 days (Houpert et al. 1995).

Compared to the cadmium concentrations achieved in pig tissues found in our previous monitoring (Svoboda et al. 2020), the values in the sheep tissues were greater. Jorhem (1999) reached a similar result in Sweden. This may be due to the fact that pigs are kept in closed spaces and have a different feed composition. As another factor, we can also mention the fact that ruminants are able to accumulate cadmium in the body at a greater intensity compared to monogastric animals because ruminants have flora in their digestive tract that produce phytase enzymes. These enzymes are able to digest phytic acid, thereby releasing cadmium from binding to phytates and, thus, increasing the absorption capacity of cadmium (Zacharias et al. 2003).

In comparison with the values found when monitoring the cadmium content in the tissues of ruminants in the Czech Republic by Drapal et al. (2021), the values obtained in our study were higher in sheep.

A similar result was also reached by the authors in Lebanon, Obeid et al. (2016). These authors state that this difference can be explained by several factors such as the size of the animals, the distribution characteristics of the cadmium in the organism as well as different eating habits. An important factor may also be the fact that sheep are mostly kept outside on pastures, while cows are more often kept in closed spaces.

The main purpose of our monitoring was to determine the content of cadmium in the tissues of sheep, primarily due to concerns for the health of the human population consuming sheep products.

For human consumption, the European Com-mission (2023) has established maximum limits (MLs) for the cadmium content of 0.050 mg.kg^–1^ in the muscle, 0.50 mg.kg^–1^ in the liver, and 1 mg.kg^–1^ in the kidneys.

In our study, the maximum limit for human consumption was not exceeded in any of the samples in the lambs, but was exceeded in 5 of 33 liver samples and in 12 of 33 kidney samples in the adult sheep.

No statistically significant trend of a decrease in the cadmium content in lamb and sheep tissues was found during the observed time period. We can sum up that there is an evident need for further monitoring of the cadmium concentration in lamb and sheep tissues in the Czech Republic.

The results of our monitoring show that a higher content of cadmium can be expected in internal organs, i.e., the liver and kidneys. A higher cadmium content in the tissues is detected in adult sheep compared to younger categories, i.e., lambs. From the point of view of cadmium intake by human consumers, there is less risk in case of using muscle tissues. Given that the maximum limit for cadmium in kidney tissues was exceeded in several cases, it is necessary to continue this monitoring.
